# Organ donation after medically assisted death on psychiatric grounds: an ethical analysis

**DOI:** 10.3389/fpsyt.2025.1574900

**Published:** 2025-05-23

**Authors:** Karlijn van Vlerken, Radboud Marijnissen, Rosalie Pronk, Guy Widdershoven, Sisco van Veen

**Affiliations:** ^1^ Department of Medical Ethics, Philosophy and History, Erasmus Medical Center, Rotterdam, Netherlands; ^2^ Department of Psychiatry, University Medical Center Groningen (UMCG), University of Groningen, Groningen, Netherlands; ^3^ IQ Healthcare, Radboud University Medical Center (UMC), Nijmegen, Netherlands; ^4^ Department of Ethics, Law and Medical Humanities, Amsterdam University Medical Center, Amsterdam, Netherlands; ^5^ Department of Psychiatry, Amsterdam University Medical Center, Amsterdam, Netherlands

**Keywords:** medical assistance in dying, organ donation, decision-making capacity, autonomy, stigmatization

## Abstract

Organ donation after medical assistance in dying (MAID) on psychiatric grounds is a relatively new practice that poses complex ethical challenges. This paper explores several ethical issues that are important for guiding current and future practice. While organ donation after MAID may alleviate organ shortages, it also prompts concerns regarding the instrumentalization of human life. However, it can be argued that if a patient wishes to donate, based on insight and deliberation, the person is not just regarded as a means. This implies that decision-making capacity is crucial, which requires considering the potential influence of psychiatric disorders. A further issue that can compromise decision-making is susceptibility to external pressures. Careful assessment of the patient’s decision-making capacity and the absence of external pressure are needed to avoid the stigmatization of individuals with psychiatric conditions. Further research to better understand the possible interplay between psychiatric disorders and decision-making capacity in the context of organ donation after MAID is recommended.

## Introduction

Medical assistance in dying (MAID) is a process by which a physician complies with a request from a patient to end his/her life in a situation of unbearable suffering. MAID is most often associated with terminal somatic illness, such as end-stage cancer (1), but it is also performed in cases of chronic illness. In a small but increasing number of countries, MAID is also legally permitted for patients who have psychiatric disorders as the basis for their request (2), referred to in this paper as MAID on psychiatric grounds. MAID on psychiatric grounds has been granted in Dutch jurisprudence since the 1990s and was codified with the “Termination of Life on Request and Assisted Suicide Act” in 2002. The request for MAID on psychiatric grounds must be assessed by an independent psychiatrist and an independent consultant to assess the due care criteria of the law (see **Box 1**).

Box 1 The Dutch legal framework sets out due diligence requirements that must be met for MAID to be legal (3). The statutory due care criteria state that the physician must:

- be satisfied that the patient’s request is voluntary and well-considered;
- be satisfied that the patient’s suffering is unbearable, with no prospect of improvement;
- have informed the patient about his/her situation and prognosis;
- have come to the conclusion, together with the patient, that there is no reasonable alternative in the patient’s situation;
- have consulted at least one other independent physician who must see the patient and give a written opinion as to whether the due care criteria set out above have been fulfilled; and
- have exercised due medical care in terminating the patient’s life or assisting in the patient’s suicide.

The request for MAID has to be assessed by a second independent specialist before the request can be realized. A physician is required to confirm the patient’s consent to MAID right before it is performed by explicitly asking the patient whether he/she wants to continue the procedure.

MAID followed by organ donation is legally possible in the Netherlands, as the Dutch law on organ donation and the aforementioned Termination of Life on Request and Assisted Suicide Act do not prohibit organ donation following euthanasia. Organ donation after MAID is also permitted in Belgium, Canada, and Spain (4). It is important to note that the laws that regulate the practices of organ donation and MAID differ in these countries (5). The Dutch guidelines for organ donation after MAID formulate specific criteria (see **Box 2**).

Box 2 Organ donation after MAID is possible if the patient:

- initiates the request himself/herself (the request does not come from the individuals with whom the patient has a therapeutic relationship),
- is able to take note of unbiased information about organ donation after euthanasia,
- has made an informed decision, based on the correct information about the consequences, and
- has made this decision free from external pressure and feels free to withdraw permission at any time.

The Dutch guidelines on organ donation following MAID, issued by the Dutch Transplantation Foundation, outline a specific order of events regarding organ donation: only after the patient’s MAID request has been approved can the topic of organ donation be discussed at the initiative of the patient. If the patient raises the subject of organ donation before the assessment procedure is completed, the physician must postpone discussing this topic until the MAID request is granted. The assessment of MAID and organ donation requests is divided into two separate procedures, carried out by different (teams of) healthcare professionals. There are no specific guidelines for organ donation following MAID on psychiatric grounds (6).

The exclusion criteria that apply to organ donation, in general, are as follows: organ donation is precluded in cases of unknown identity of the donor, anencephaly, acute sepsis, and certain types of infectious diseases such as tuberculosis, rabies, rubella, and herpes zoster. Relative contraindications include malignancy, HIV, chronic Q fever, and unknown cause of death (6, 7). The majority of these do not apply in cases of MAID on psychiatric grounds.

One consequence of legalizing MAID on psychiatric grounds is the possibility of organ donation following MAID on psychiatric grounds. In the Netherlands, organ donation after MAID on psychiatric grounds occurred 24 times between 2012 and 2022, amounting to 28.9% of all cases in which MAID was followed by organ donation (8). Notably, there was a substantially larger percentage of patients with psychiatric conditions who donated their organs than the percentage of patients who requested MAID on other grounds, an estimated 3.8% versus 1.1%, respectively (8). One possible reason for this may be that, generally, individuals requesting MAID solely on psychiatric grounds have fewer somatic contraindications to organ donation than other patients. However, questions can be raised about how the possibility of organ donation after MAID on psychiatric grounds may intersect with the specific circumstances of individuals with psychiatric conditions, potentially influencing their decision-making process. For instance, empirical research suggests that the capacity for giving consent to organ donation in patients diagnosed with schizophrenia spectrum disorders may be affected by factors related to the disorder, such as positive and negative symptoms, and that the capacity to consent to research does not always translate into the capacity to give consent to organ donation (9).

The possibility of organ donation after MAID remains a controversial topic, as there is no consensus in the current literature as to what ethical safeguards and regulations should be in place (5). Organ donation after MAID, specifically in the context of psychiatric suffering, further raises complex ethical issues that, to our knowledge, have not been explored in the academic literature. This article aims to highlight several ethical arguments regarding organ donation after MAID on psychiatric grounds, pertaining to saving lives, dignity, decision-making capacity, and vulnerability. While some of the arguments can also be applied to the independent practices of organ donation and MAID on psychiatric grounds, the combination of the two introduces an additional layer of complexity that may offer unique ethical challenges while also providing an opportunity to advance the discussion surrounding both practices. This paper will explore some key questions in relation to various ethical aspects, such as the following: what are the potential societal and individual benefits of organ donation after MAID on psychiatric grounds? What are the specific complexities and vulnerabilities that psychiatric suffering brings to decision-making capacity in this context?

## Saving lives

One argument in favor of organ donation after MAID on psychiatric grounds is that it can potentially save the lives of patients who would otherwise die while waiting for a suitable donor. This argument is often applied regarding organ donation in general. Organ transplant shortages are a significant problem worldwide, leading to long waiting lists for patients in need of transplants. The US Organ Procurement and Transplantation Network estimates that over 100,000 individuals are on the waiting list to receive an organ transplant and that every day, 17 patients die in the United States while waiting (10). Similar numbers can be found in Europe, with an average of 21 patients dying every day while waiting for an organ transplant in 2022 (11). As organ donation after MAID is carried out under controlled clinical circumstances, there is a higher chance of successfully transplanting organs as opposed to death outside of a clinical setting. In addition, organs from one donor may benefit multiple recipients. In this way, organ donation after MAID on psychiatric grounds has the potential to reduce suffering and improve the overall health of the population.

However, it can be argued against this line of reasoning that it is an instrumental view of a person which does not respect the intrinsic dignity of each individual. Individuals appear to be essentially viewed as a means to an end, in this case as a source of organs for transplantation. This would imply that the practice of organ donation after MAID goes against the Kantian rule that individuals should not be merely used as a means to an end. Treating or viewing individuals in an instrumental way in the context of organ donation after MAID can raise moral and societal questions about the value of human dignity. Critics may argue that it potentially erodes the ethical foundations of medical practice that prioritize the dignity of patients even after death.

However, this counter-argument not only makes organ donation after MAID problematic in psychiatry but also undermines the practice of organ donation in general. Moreover, it may be questioned whether, in the practice of organ donation, a person is merely regarded as a means to an end. If the donation is based on someone’s free and autonomous decision to allow his/her organs to be used, it can be said that the person is treated not only as a means but also as an end (12). By permitting organ donation after MAID on psychiatric grounds, persons who have made a well-considered request to end their lives through MAID and who opt for the donation of their organs with insight into the situation and after deliberating on the consequences can exercise their autonomy until the very end. This argument will be further explored in the next section.

## Decision-making capacity

Allowing organ donation after MAID can be seen as allowing a patient to realize his/her “last wish”. For patients, altruism can be an important drive for organ donation after MAID, as donation can bring hope and healing to others. There may be a sense of relief in having the possibility to donate organs after MAID, and it can be a potential source of meaning-making in the context of severe suffering (13, 14). In contrast to organ donation after circulatory death, organ donation after MAID allows for a conscious patient to decide if, when, and how he/she wants to donate his/her organs (15). An important condition for this is that the patient is competent to make such a decision, or, in other words, that the patient has adequate decision-making capacity.

Previous research has developed various concepts of competence. One well-known concept focuses on cognitive abilities. This approach entails that to be considered competent, a patient should be able to communicate a decision, comprehend relevant information, appreciate the situation and its consequences, and reason about possible options (16). An alternative concept of competence emphasizes the relevance of practical rationality. This implies that the patient should be emotionally engaged with the decision and understand its relevance in the light of what matters in living a good life (17).

In the literature, several concerns have been raised regarding the competence of psychiatric patients in the context of end-of-life decisions. For example, patients diagnosed with severe depression can have anomalous experiences of temporality, which alter their appreciation of the situation and can thus undermine their decision-making capacity (18). Another factor that can affect decision-making capacity is patients’ perceived burdensomeness or the perception that they are a burden to others or to society at large, which is believed to play a causal role in some suicides (19). It has been suggested that perceived burdensomeness may compromise a patient’s decision-making capacity regarding a request for MAID (20).

Concerns have also been raised regarding the possible role of complex interpersonal psychodynamic processes, which can, for instance, be present in some personality disorders, in the request for MAID, and the assessment of this request (21). In Dutch practice, the possible influence of a psychiatric disorder on the request for MAID is recognized, and decision-making capacity regarding a request for MAID on psychiatric grounds has to be assessed by an independent psychiatrist (22).

It is important to note that determining whether someone has adequate decision-making capacity always pertains to a specific decision in a specific context at a specific time. This implies that persons may have the capacity to make a request for MAID, as they understand the nature of their suffering and the consequences of MAID, but may lack the capacity to donate organs, as they have unrealistic views, for instance, feeling that donation is the only way to make their life worthwhile. It is conceivable that nihilistic tendencies may result in selfless actions, such as proposing organ donation, or even as maladaptive variants of altruism, such as excessive self-sacrifice, which can be a part of an agreeable personality structure (23). The aforementioned factors may affect decision-making capacity regarding organ donation after MAID on psychiatric grounds, for instance, by compromising practical rationality, including the ability to balance the values of altruism and care for oneself. On the basis of these arguments, it can be justified that the competence regarding decisions around organ donation of psychiatric patients is investigated independently of the competence required for a request for MAID. It must be noted, however, that the likelihood of a situation in which a patient is competent to request MAID and not to decide about organ donation is not very high. Moreover, problems regarding decision-making capacity should not automatically be assumed in the case of a psychiatric disorder. Subjects who do not suffer from psychiatric illness may also have an anomalous understanding of their situation (24), and pointing to such presumed vulnerabilities, may contribute to the stigmatization of individuals suffering from psychiatric illness.

## External pressure

In addition to issues regarding decision-making capacity, individuals who are involved in a MAID request may be vulnerable to suggestions from outside sources. External forms of undue influence, for example, from peers, the media, or society, may aggravate a susceptibility that can be associated with psychiatric illness. For example, a patient can be affirmed in his/her maladaptive altruistic tendencies if there is a social discourse in which organ donation after MAID is encouraged. In addition, the practical aspects of organ donation after MAID may compromise freedom of choice. Because of the need for controlled medical circumstances to transplant organs, in the majority of cases, organ donation after MAID is performed in medium or intensive care units to maximize the chance of successful organ transplantation (8). Although patients should be able to opt out of the organ donation procedure at any moment, the presence of medical staff, being in a hospital room, and being transported by an ambulance may all compromise a patient’s ability to withdraw from either MAID or the organ donation process. Moreover, the structured environment in which the procedure of MAID before organ donation takes place can limit a patient’s sense of privacy and make it more challenging for him/her to reflect on his/her decisions independently, which can be a potentially negative aspect of the practice. However, the presence of healthcare professionals may also provide a source of support for the patient in his/her decision-making process until the very end, for example, by carefully observing the patient and asking crucial questions (25).

External influence is not *a priori* problematic, however. Care ethicists emphasize the idea that autonomy is not merely an individual characteristic of a person, but can only be developed in relation to others (26). In the case of MAID on psychiatric grounds, the Dutch guideline stipulates that relatives should be involved in the process (22). This also has consequences for decisions regarding organ donation after MAID. Regardless of the nature of the underlying condition, the procedure that organ donation entails can be a cause of distress for relatives. The patient dies in unfamiliar surroundings, and the next of kin has only a limited amount of time to grieve over the body of the deceased patient. However, organ donation after MAID on psychiatric grounds could also help to reduce the emotional burdens faced by the patients’ relatives, thereby supporting them in their grief. This idea is endorsed by two case studies that suggest that the burden of organ donation after MAID is minimal for some patients and their relatives and that it may even be helpful. Relatives reported feeling proud that the patient was able to potentially alleviate the suffering of others (25).

## Discussion

Organ donation after MAID on psychiatric grounds is legally allowed and medically possible in the Netherlands. The unique circumstances surrounding organ donation after MAID on psychiatric grounds raise complex ethical issues that require careful consideration. It can be argued that organ donation following MAID on psychiatric grounds potentially saves the lives of those waiting for organ transplants. However, this should not lead to an instrumental approach that harms the dignity of individuals. In the context of organ donation after MAID on psychiatric grounds, specific challenges regarding decision-making capacity can be identified, such as the anomalous experience of temporality, perceived burdensomeness, and complex interpersonal psychodynamic processes. Concerns may also arise about the possibility of broader societal pressures to develop, which could subtly influence patients to feel steered toward a specific course of action.

In line with previous literature (27–30), this article further outlines some of the ethical challenges relating to autonomy and decision-making capacity that can arise when the processes of organ donation and MAID become intertwined. The previously mentioned specific factors relating to psychiatric suffering can interfere with the decision-making process, which in turn can further undermine an autonomous decision. As an ethical safeguard, current Dutch guidelines separate the procedures of MAID and organ donation, and the request for organ donation can only be discussed at the patient’s initiative after the request for MAID has been evaluated positively by the physician, in line with the consensus in the literature (5). We argue that in some cases, it may be needed to assess the decision-making capacity of the organ donation request separately from the request for MAID in order to ensure that both choices are made voluntarily and free from undue influence. For the assessment of the decision-making capacity to donate, we suggest paying particular attention to the motivation of the patient and whether it is based on adequate retrieval of information, shows adequate emotions, and expresses values that fit into the patient’s life (17).

Although authors have argued that the separation of organ donation from the MAID procedure can, for a large part, be ensured within the administrative and logistical measures that are being taken before and during the medical procedure (28), the question remains whether it is possible for patients and physicians to differentiate between the two decision-making processes (30). With that being said, efforts should be put into the prevention of potential stigmatization of individuals suffering from psychiatric illness. Further academic debate and research are needed to investigate the extent to which the decision to donate organs can be influenced by the choice for MAID and vice versa, and whether situations occur in which an individual’s request for MAID is considered to be competent, but his/her decision regarding organ donation is not.

Organ donation after MAID on psychiatric grounds is a complex practice that entails ethical issues. As the practice is relatively new and rare, this article aims to stimulate further debate and deliberation on the ethical arguments that can be raised in this specific practice. While organ donation after MAID on psychiatric grounds may offer benefits in dealing with organ shortages and honoring patient autonomy, it also can raise questions regarding the risk of instrumentalizing human life and possible problems concerning decision-making capacity related to psychiatric illness. In the context of organ donation after MAID on psychiatric grounds, specific challenges regarding decision-making capacity can be identified, such as the anomalous experience of temporality, perceived burdensomeness, and complex interpersonal psychodynamic processes. If these factors interfere with the decision-making process, an autonomous decision can be undermined. There is a need for a careful assessment of decision-making capacity without stigmatizing individuals with psychiatric illnesses. Also, the possibility of external pressure has to be critically examined. More empirical and qualitative research is needed to investigate the relationship between psychiatric disorders and decision-making capacity regarding organ donation after MAID, focusing on the underlying motivations and potential factors that may influence and enhance an autonomous decision-making process regarding organ donation after MAID on psychiatric grounds.

## Data Availability

The original contributions presented in the study are included in the article/supplementary material. Further inquiries can be directed to the corresponding author.
